# Analysis of the effects of acetyl tributyl citrate on bone cancer based on network toxicology and molecular docking

**DOI:** 10.3389/fmed.2025.1613657

**Published:** 2025-06-23

**Authors:** Lujun Jiang, Juncheng Shen, Jinghong Yang, Zi Wang, Lian Tang, Yuqi Li, Jialin Liu, Zhong Li, Yanshi Liu

**Affiliations:** ^1^Department of Orthopedics, Affiliated Hospital of Southwest Medical University, Luzhou, China; ^2^Sichuan Provincial Laboratory of Orthopaedic Engineering, Luzhou, China; ^3^Stem Cell Immunity and Regeneration Key Laboratory of Luzhou, Luzhou, China; ^4^Department of Urology, Affiliated Hospital of Southwest Medical University, Luzhou, China; ^5^Department of Oral Implantology, The Affiliated Stomatological Hospital, Southwest Medical University, Luzhou, China; ^6^Luzhou Key Laboratory of Oral and Maxillofacial Reconstruction and Regeneration, Luzhou, China

**Keywords:** acetyl tributyl citrate, bone cancer, network toxicology, molecular docking, mechanism

## Abstract

**Background:**

Acetyl tributyl citrate (ATBC) is a widely used environmental plasticizer that has raised concerns regarding its potential health effects, particularly its role in cancer development. Although ATBC is generally considered to have a safer profile compared to traditional phthalate-based plasticizers, research on its association with bone cancer remains limited. The aim of this study is to elucidate the complex effects of Acetyl tributyl citrate (ATBC) on bone cancer and to unravel the potential molecular mechanisms by which environmental pollutants influence the disease process.

**Methods:**

This study utilized multiple online databases to identify target genes associated with ATBC and bone cancer. Initially, protein–protein interaction (PPI) analysis and visualization of the intersecting genes were performed. Subsequently, gene ontology (GO) and Kyoto Encyclopedia of Genes and Genomes (KEGG) functional enrichment analyses were conducted to explore the underlying mechanisms connecting the two conditions. Finally, molecular docking was employed to validate the interactions between these compounds and their respective targets.

**Results:**

Using the CHEMBL, SwissTarget Prediction, and TargetNet databases, we screened 193 genes associated with ATBC. Additionally, we identified 4,439 genes related to bone cancer through the GeneCards, OMIM, and TTD databases, resulting in 73 intersecting genes. After rigorous refinement utilizing the STRING platform and Cytoscape software, we identified five core targets: STAT3, EGFR, MMP9, MAPK1, and MMP2. Functional enrichment analysis indicated that the core targets of ATBC’s influence on bone cancer are primarily involved in the regulation of apoptosis, carcinogenesis, and cellular proliferation, among other biological processes. Finally, molecular docking simulations conducted with AutoDock confirmed robust binding interactions between ATBC and these core targets, thereby enhancing our understanding of their interactions.

**Conclusion:**

This study underscores the potential carcinogenic effects of ATBC in bone cancer, identifying key targets such as STAT3, EGFR, MMP9, MAPK1, and MMP2. The findings indicate that ATBC may facilitate the progression of bone cancer by targeting essential signaling pathways and remodeling the tumor microenvironment. This emphasizes the necessity for further research into the environmental risks associated with this plasticizer.

## 1 Introduction

Acetyl Tributyl Citrate (ATBC) is an environmentally friendly plasticizer widely utilized in various industries, including plastics, rubber, coatings, and food packaging. Due to its low toxicity and biodegradability, ATBC is regarded as an ideal alternative to traditional phthalate-based plasticizers (1). With growing concerns regarding the environmental and health risks associated with phthalate plasticizers—known to disrupt endocrine function and pose long-term health threats—ATBC has become increasingly preferred for use in products that may come into contact with food and sensitive environments (2, 3). However, despite its safety advantages over phthalate plasticizers, the potential long-term effects of ATBC on human health have raised significant concerns (4). Recent studies indicate that ATBC may adversely affect organisms through mechanisms such as interference with metabolic pathways, induction of inflammatory responses, or disruption of cellular homeostasis. For instance, animal studies have demonstrated that exposure to ATBC can lead to obesity and fatty liver phenotypes, suggesting its potential impact on multi-organ function through lipid metabolism disruption (5). Nevertheless, most research has concentrated on the general metabolic disruptions caused by ATBC, with limited investigation into its specific role in diseases such as bone cancer.

Bone cancer, while rare, represents a highly aggressive malignancy that presents significant medical challenges due to its complex and poorly understood etiology. The development of bone cancer is closely associated with genetic mutations, epigenetic alterations, microenvironmental imbalances, and the activation of critical signaling pathways (6, 7). Pathways such as Wnt/β-catenin and PI3K/AKT play crucial roles in tumorigenesis by regulating essential processes, including cell proliferation, survival, and metastasis. The progression of bone cancer, particularly osteosarcoma, is marked by an imbalance between osteoblasts and osteoclasts, leading to abnormal bone remodeling and uncontrolled tumor growth (8, 9). While much research has focused on the relationship between heavy metals, organic pollutants, and bone cancer, limited investigation has been conducted into the role of plasticizers, such as ATBC, in the development of bone cancer. As a relatively new environmental contaminant, the accumulation of ATBC in bone tissue and its effects on the osteoblast–osteoclast balance remain inadequately explored. This research gap underscores the necessity for a comprehensive investigation into the carcinogenic potential of ATBC, particularly within the context of bone cancer, which continues to be an underexplored area in toxicology and cancer research.

Network toxicology is an emerging approach that integrates high-throughput omics data with bioinformatics analysis, facilitating the systematic identification of chemical toxicity targets and their interaction networks. This method provides a robust strategy for elucidating the complex mechanisms underlying toxicity (10). For example, combined transcriptomic and metabolomic analyses have successfully revealed metabolic pathway reprogramming in the liver under ATBC exposure (5).

In addition, molecular docking has become a valuable computational tool for understanding how environmental chemicals interact with key proteins in the body (11). Molecular docking simulations not only predict the binding affinity between chemical compounds and target proteins but also visualize and quantify the interactions that may result in toxic effects (12, 13). Utilizing molecular docking technology, the binding patterns of ATBC with key proteins can be predicted, allowing for the verification of its direct targets and elucidating the potential mechanistic pathways through which ATBC contributes to the pathogenesis of bone cancer (14).

This study aims to construct a toxic interaction network for ATBC using network toxicology, identify key targets and pathways associated with bone carcinogenesis, and validate the interactions of ATBC with core proteins through molecular docking. The findings will not only enhance our understanding of the molecular mechanisms of ATBC but also provide a scientific foundation for future safety evaluations and the development of sustainable alternatives to harmful chemicals in consumer and industrial products.

## 2 Methods

### 2.1 Collection of ATBC target genes

The standard structure and SMILES symbol of ATBC were determined by querying “acetyl tributyl citrate” in the PubChem database.^1^ Using this information, we retrieved the potential target of ATBC from the CHEMBL database,^2^ used the keyword “acetyl tributyl citrate,” and narrowed the search scope to “*Homo sapiens*.” Subsequently, the Canonical SMILES codes of these compounds were uploaded to the SwissTarget Prediction database^3^ and the TargetNet database.^4^ The target data collected from these databases was then consolidated, duplicates were removed, and the target names were standardized using the Uniprot database.^5^ This process culminated in the establishment of a comprehensive target database on ATBC.

### 2.2 Selection of target networks related to bone cancer

Utilizing the GeneCards database,^6^ OMIM database,^7^ and TTD database,^8^ we identified relevant targets pertinent to “bone cancer” by employing the keyword search. Genes were selected based on the following criteria: relevance score greater than 10 in GeneCards and all genes in OMIM and TTD. We then used a Venn diagram analysis to integrate the genes screened from the 3 databases to obtain a final set of bone cancer-related genes. Furthermore, we employed a Venn diagram analysis to pinpoint the common potential targets shared between the ATBC targets and bone cancer targets, designating the overlapping section as the potential targets of ATBC that specifically impact bone cancer.

### 2.3 Protein interaction network construction and core target screening

We input the cross genes of potential targets of ATBC affecting bone cancer into the STRING database,^9^ and limit the species to “*Homo sapiens*.” Set the “minimum required interaction score” to “medium confidence >0.4” for analysis. The results were then visualized using Cytoscape 3.10.3 software,^10^ which enabled the construction of a protein interaction network.

### 2.4 Gene function and pathway enrichment analysis of the target protein

We utilized the DAVID online tool^11^ for Gene Ontology (GO) analysis and Kyoto Encyclopedia of Genes and Genomes (KEGG) pathway analysis to identify functional annotations and pathway enrichment related to potential genes. Following this, we performed visual analysis on the Bioinformatics online platform^12^ to interpret and present the results of the GO and KEGG analyses.

### 2.5 Molecular docking

PDB format files for the key genes were retrieved from the RCSB database,^13^ and molecular structure files for the ATBC were downloaded from the PubChem database. The molecular docking protocol was systematically conducted as follows: Initially, all solvent molecules and non-essential ligands were removed from the five target protein structures using PyMOL 2.6.0. This was followed by structural optimization and export in PDB format. Subsequently, the Getbox Plugin was utilized to calculate the three-dimensional grid box parameters that define the active sites, with particular emphasis on including key catalytic residues. Following this preparation, both the processed macromolecular structures and small molecule ligands were converted to PDBQT files using AutoDock Tools 1.5.6. The docking simulations were then conducted using AutoDock Vina 1.1.2. Finally, the molecular docking results were visualized with PyMOL. The binding score was employed to evaluate the interaction between ligands and receptors: a binding score of less than 0 kcal/mol indicates spontaneous binding, while a binding score of less than −5 kcal/mol suggests stable binding.

## 3 Results

### 3.1 Identification of targets of ATBC affecting bone cancer

**Figure 1:**
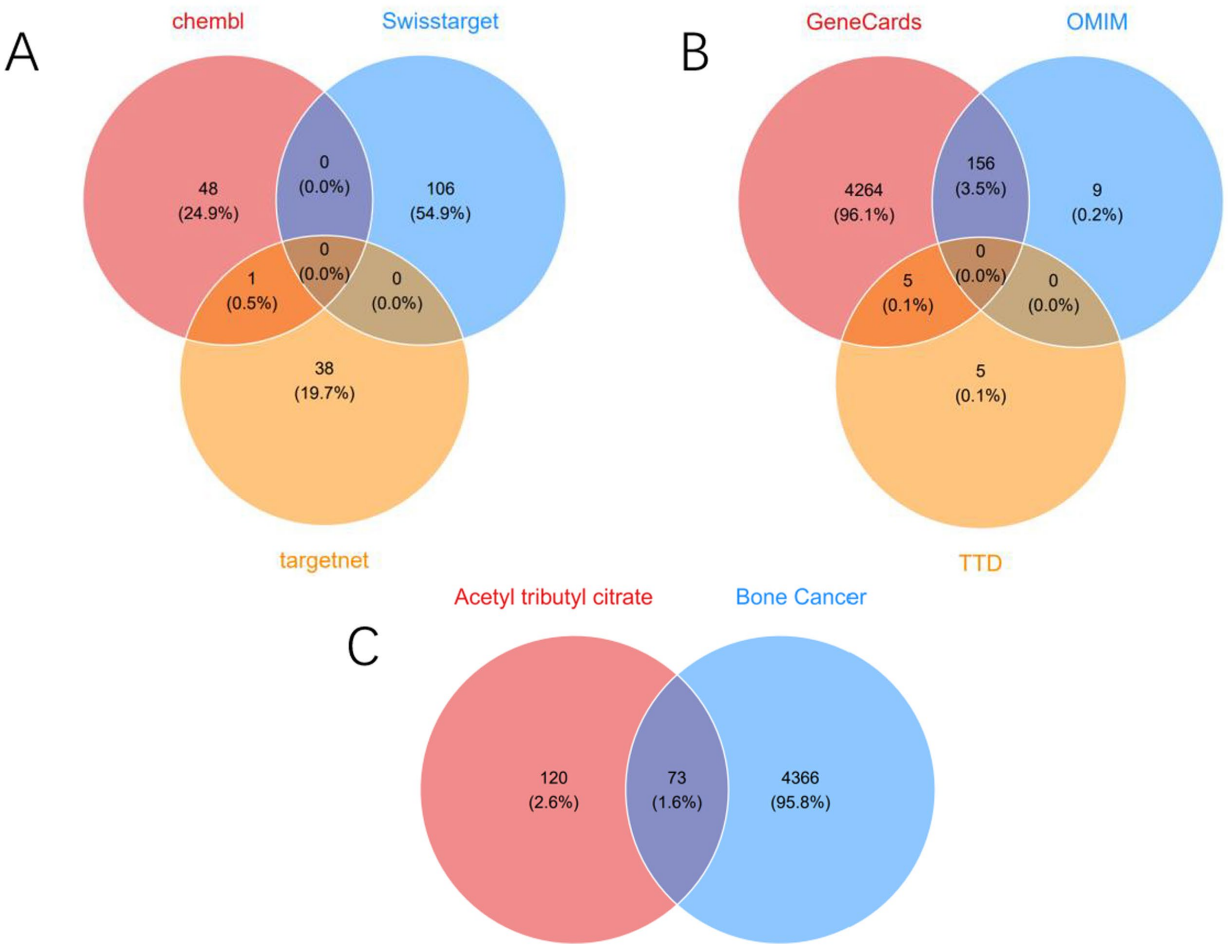
**(A)** Venn diagram of potential targets of ATBC. **(B)** Venn diagram of potential targets of bone cancer. **(C)** Venn diagram of the intersecting targets of ATBC with bone cancer.

By integrating screening results from the CHEMBL, SwissTarget Prediction, and TargetNet databases, we obtained 193 targets related to ATBC (Figure 1A). Subsequently, utilizing the comprehensive information available in the GeneCards, OMIM, and TTD databases, we identified 4,439 targets strongly correlated with bone cancer (Figure 1B). Ultimately, we identified 73 targets that may play a significant role in ATBC-induced bone cancer (Figure 1C). Based on the identification of these targets, we subsequently conducted a Protein–Protein Interaction (PPI) network analysis to further elucidate the interactions among these targets.

### 3.2 Interaction network of potential targets and acquisition of core genes

**Figure 2:**
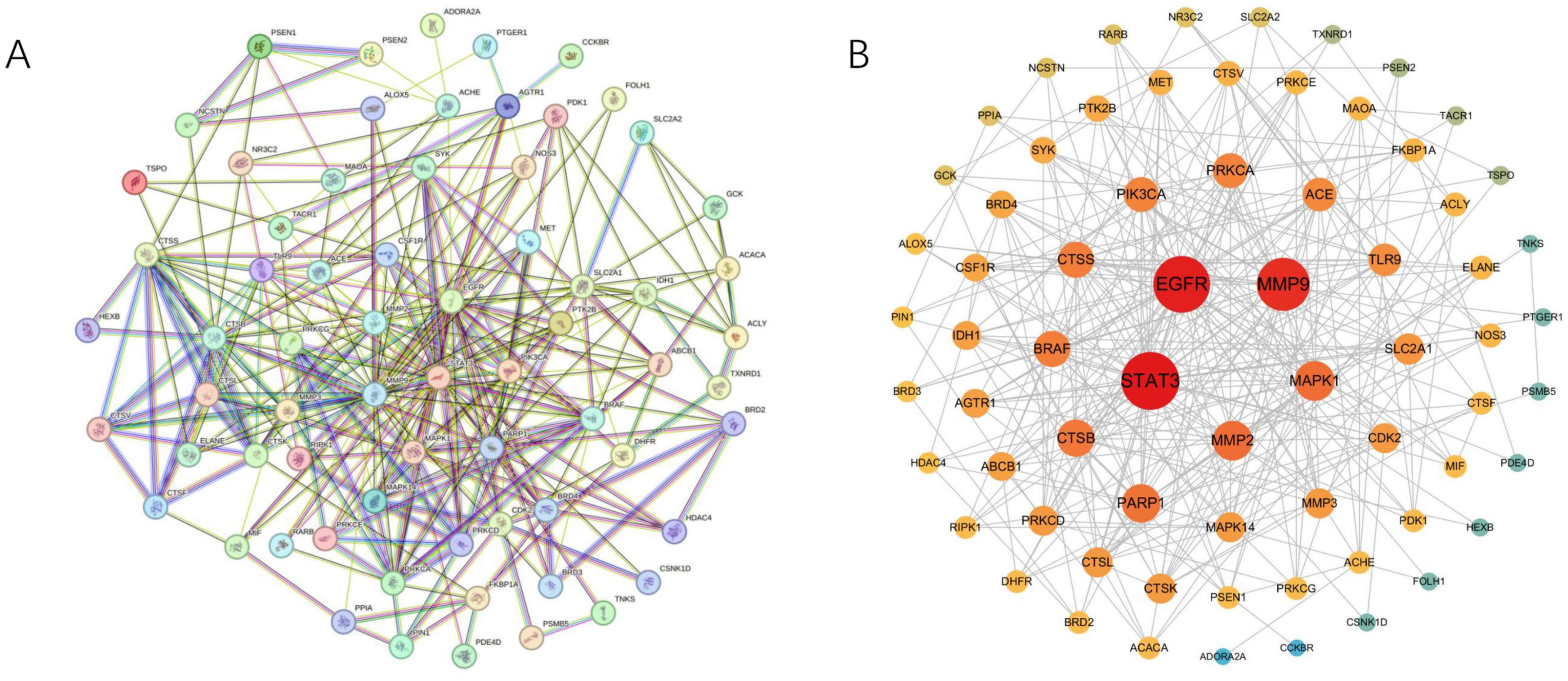
**(A)** The PPI network was constructed using the STRING database with a confidence score threshold of ≥0.4. Nodes represent proteins, and edges indicate interactions between them. The network highlights the functional associations among ATBC-bone cancer-related targets. **(B)** The PPI network was further visualized and analyzed using Cytoscape 3.10.3. Nodes are colored and sized according to their degree values, with darker colors and larger circles indicating stronger interactions.

We imported 73 intersecting target genes between ATBC and bone cancer into the STRING database for protein–protein interaction (PPI) analysis, establishing a confidence score threshold of ≥ 0.4. After filtering out isolated targets, 68 ATBC-bone cancer-related targets remained (Figure 2A). We subsequently utilized Cytoscape 3.10.3 software to visualize the PPI network. In this network, targets are ranked by Maximum Clique Centrality (MCC); darker colors and larger circles indicate stronger interactions with other proteins (Figure 2B). This enhanced visualization encapsulates the complex functional relationships and interdependencies within this select group, providing a clear and concise representation of their interconnectedness. Notably, among these core targets, STAT3, EGFR, MMP9, MAPK1, and MMP2 were identified as the top five targets based on MCC values, serving as the hub targets of ATBC-induced bone cancer.

### 3.3 Go and KEGG analyze potential targets

**Figure 3:**
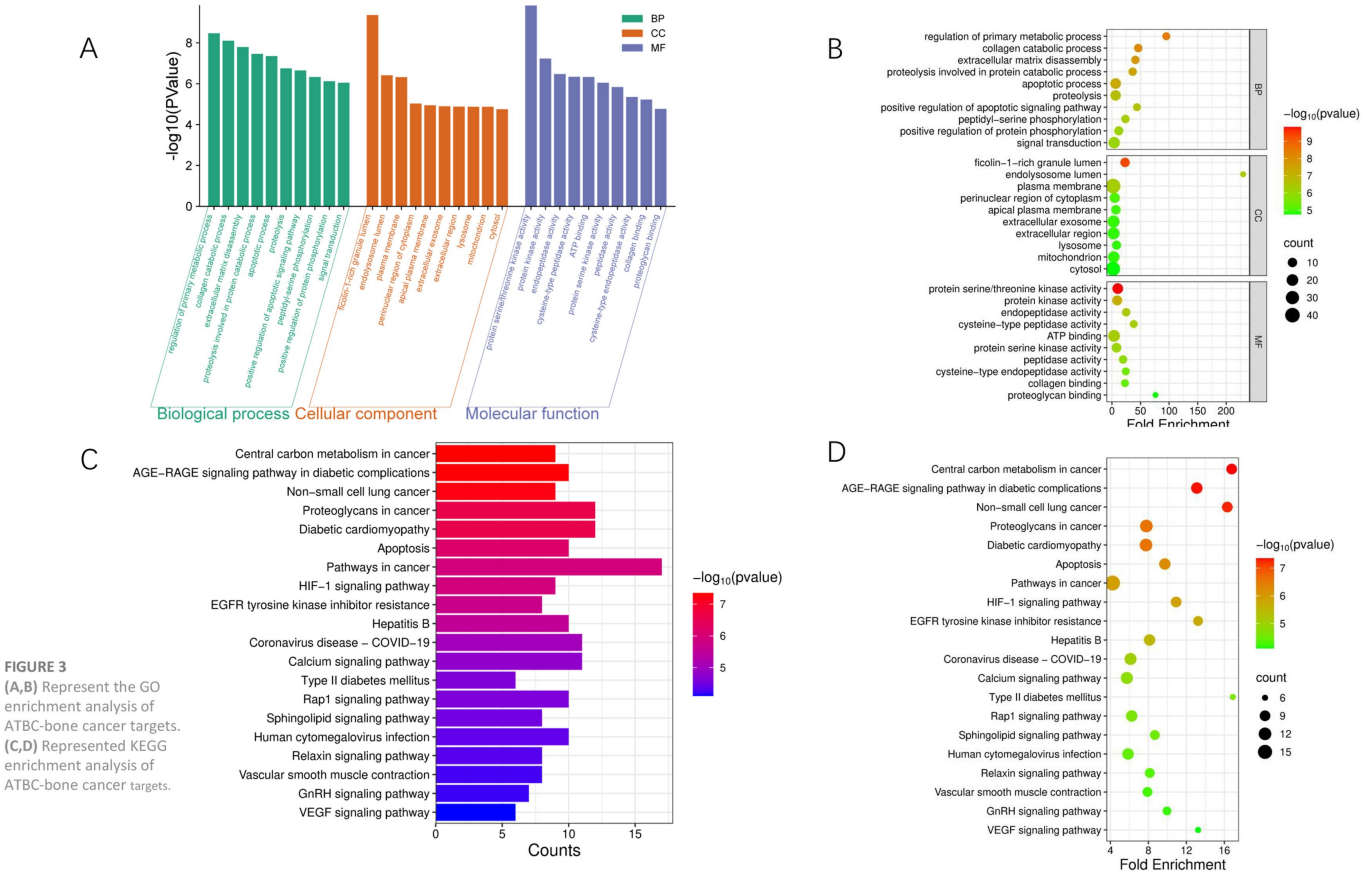
**(A,B)** Represent the GO enrichment analysis of ATBC-bone cancer targets. **(C,D)** Represented KEGG enrichment analysis of ATBC-bone cancer targets.

Through Gene Ontology (GO) and Kyoto Encyclopedia of Genes and Genomes (KEGG) analysis, we further explored the potential functions and mechanisms of action of these targets in bone cancer. In this study, 73 key targets were analyzed for Gene Ontology (GO) enrichment using the DAVID online tool, revealing 215 biological processes (BP), 55 cellular components (CC), and 104 molecular functions (MF) within the GO entries. Figure 3 presents the top 10 entries of -logP values for BP, CC, and MF. The GO functional enrichment analysis indicates that these genes are primarily involved in biological processes such as tissue remodeling and apoptosis signaling. Additionally, the results of the KEGG pathway analysis suggest that the gene set is significantly implicated in the metabolic reprogramming of tumor cells, thereby supporting the energy requirements for rapid cancer cell proliferation. Furthermore, these genes may regulate lung cancer progression or promote metastasis through proteoglycan-mediated cell adhesion and migration, indicating that the regulation of tumor survival by the hypoxic microenvironment may drive angiogenesis or metabolic adaptation. Collectively, these findings suggest that ATBC may influence the onset and progression of bone cancer through the modulation of apoptosis, cancer-related pathways, and the hypoxic response.

### 3.4 Molecular docking verification

**Figure 4:**
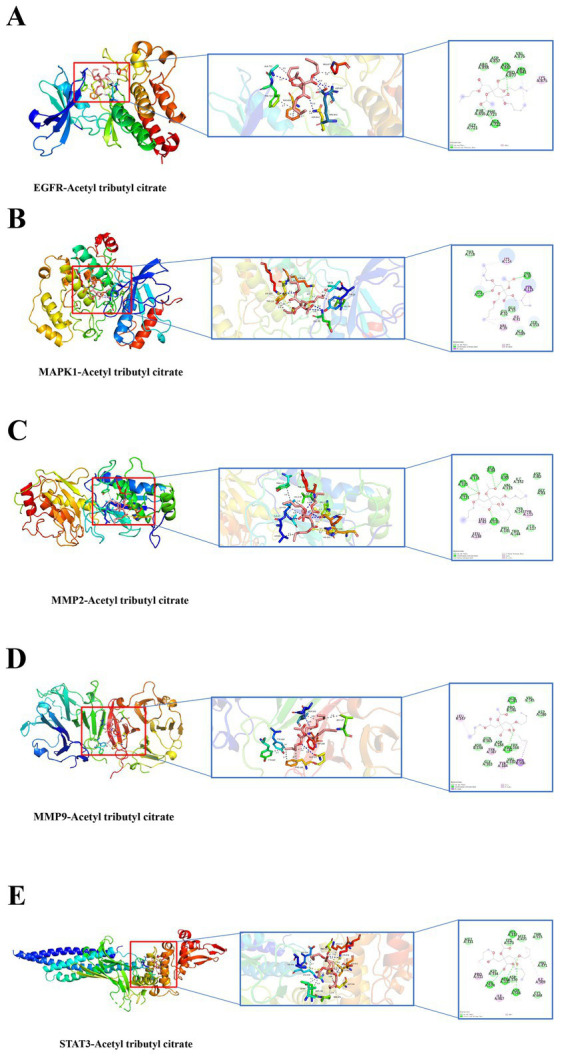
Molecular docking in each target protein with the ATBC. **(A)** ATBC and EGFR; **(B)** ATBC and MAPK1; **(C)** ATBC and MMP2; **(D)** ATBC and MMP9; **(E)** ATBC and STAT3.

**Figure 5:**
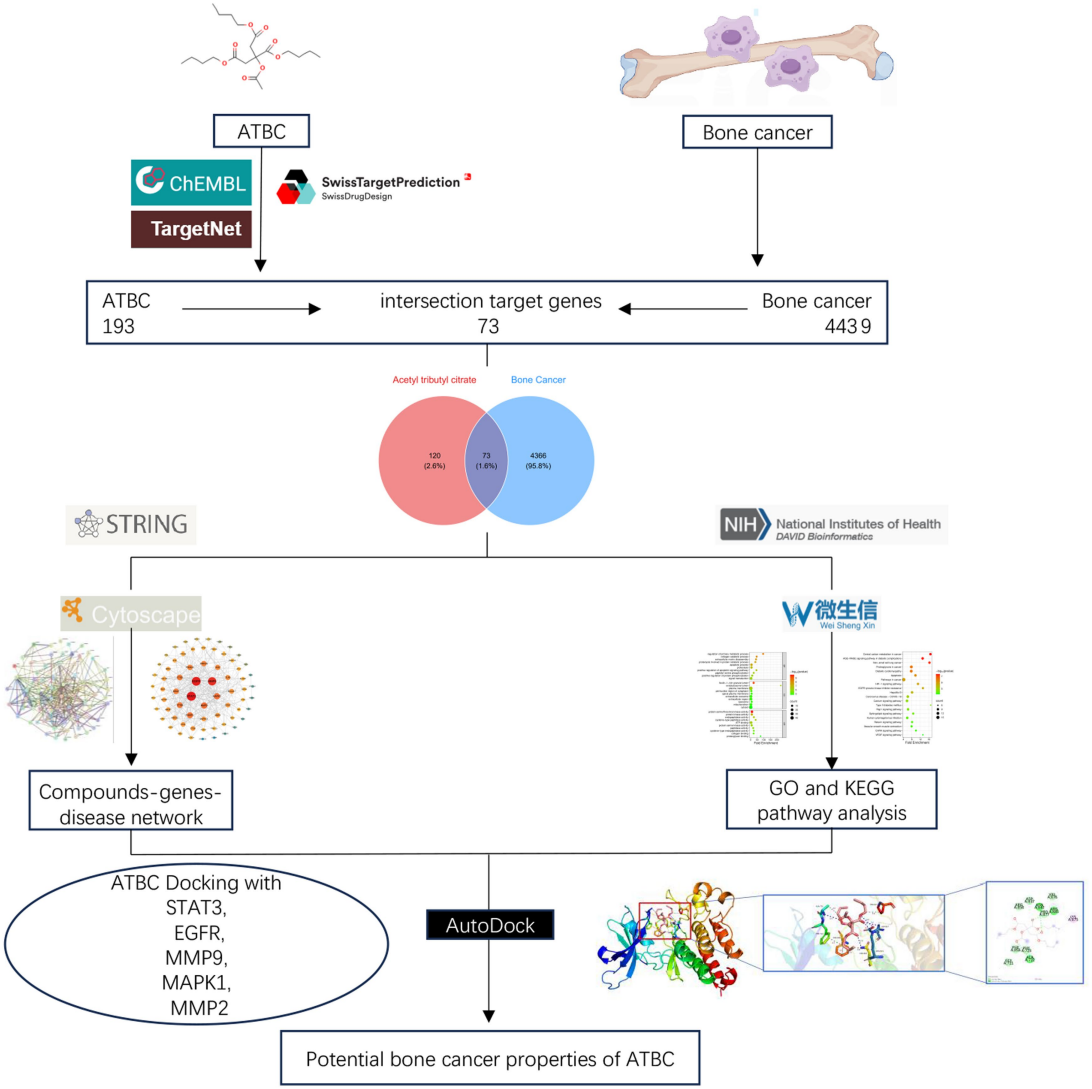
Flowchart of the whole experiment.

Molecular docking simulations were performed between ATBC and the target proteins EGFR, MAPK1, MMP2, MMP9, and STAT3. The binding stability of these complexes is assessed based on the magnitude of the binding scores (Table 1). A negative binding score indicates the formation of a stable complex, while a lower binding score suggests a higher likelihood of ligand-receptor interaction. The results indicated that ATBC exhibited a binding score of −5.9 kcal/mol for EGFR. The binding of ATBC to EGFR was facilitated by van der Waals interactions with residues PHE856, PHE723, GLY721, ARG858, ASP837, PRO877, and VAL876, as illustrated in the 3D and 2D mappings. Furthermore, ATBC demonstrated stable binding to EGFR through the formation of hydrogen bonds with ALA722, ASN842, and ARG841. Additionally, alkyl interaction bonds with LYS875 residues contributed to the stable binding of ATBC to EGFR (Figure 4A). The binding score of ATBC for MAPK1 was −5.1 kcal/mol. Van der Waals interactions involving residues THR118, GLU33, GLY32, ALA189, and SER153 mediated the binding of ATBC to MAPK1, as shown in both 3D and 2D maps. Moreover, ATBC exhibited stable binding to MAPK1 by forming hydrogen bonds with LYS117 and TYR30. Furthermore, the binding of ATBC to MAPK1 was facilitated by *π*-alkyl and alkyl interaction bonds with VAL39, ILE31, and LYS114 residues, as well as *π*-sigma interaction bonds with TYR113 (Figure 4B). ATBC displayed a binding score of −6.2 kcal/mol to MMP2. The interaction of ATBC with MMP2 was mediated by van der Waals interactions with residues PRO141, THR144, LEU117, TYR143, VAL118, ASP80, and GLY81, as depicted in 3D and 2D maps. Furthermore, ATBC exhibited stable binding to MMP2 through the formation of hydrogen bonds with ALA140, HIS121, HIS125, HIS131, ALA84, LEU83, and ILE142. Additionally, the stable binding of ATBC to MMP2 was associated with *π*-alkyl and alkyl interaction bonding to LEU82, LEU138, and TYR113 residues (Figure 4C). The binding score of ATBC for MMP9 was −5.5 kcal/mol. The favorable van der Waals interactions with residues ASP148, GLN163, ASP188, TRP168, GLY183, VAL182, PRO146, VAL145, and ASP180 facilitated the binding of ATBC to EGFR, as illustrated by both 3D and 2D mapping. Additionally, ATBC exhibited stable binding to MMP9 through the formation of hydrogen bonds with GLN181 and VAL182. Furthermore, the binding of ATBC to MMP9 was mediated by π-alkyl and alkyl interaction bonds with the LEU147, TYR187, and TYR184 residues, as well as π-Sigma interaction bonds with the PHE166 residue (Figure 4D). The binding score of ATBC to STAT3 was −5.0 kcal/mol. The interaction between ATBC and STAT3 was found to be mediated by van der Waals interactions with the residues MET331, LYS573, MET470, THR515, ASP334, ASP570, CYS468, and PRO471, as depicted in the 3D and 2D maps. Moreover, ATBC demonstrated stable binding to STAT3 by forming hydrogen bonds with LYS574, ARG335, ASP566, and HIS332. Finally, alkyl interaction bonds with the residues PRO33, ILE467, and ILE569 contributed to the stable binding of ATBC to STAT3 (Figure 4E). The flowchart of the whole experiment is shown in Figure 5.

**Table 1:** The binding scores and the binding pockets.

Target	Compound	binding score (kcal/mol)	Binding pocket
EGFR	Acetyl tributyl citrate	−5.9	--center_x -5.9 --center_y 23.4 --center_z −27.4 --size_x 30.0 --size_y 30.7 --size_z 29.9
MAPK1	Acetyl tributyl citrate	−5.1	--center_x 47.5 --center_y 33.7 --center_z 3.2 --size_x 17.8 --size_y 20.0 --size_z 20.4
MMP2	Acetyl tributyl citrate	−6.2	--center_x 27.7 --center_y 14.5 --center_z −8.0 --size_x 18.5 --size_y 19.7 --size_z 19.9
MMP9	Acetyl tributyl citrate	−5.5	--center_x -25.1 --center_y -45.0 --center_z −5.0 --size_x 20.5 --size_y 17.5 --size_z 17.8
STAT3	Acetyl tributyl citrate	−5.0	--center_x 11.9 --center_y 35.6 --center_z 20.5 --size_x 20.9 --size_y 14.6 --size_z 17.1

## 4 Discussion

This study systematically investigates the molecular mechanisms underlying the effects of acetyl tributyl citrate (ATBC) on bone carcinogenesis through an integrated network toxicology and molecular docking approach. We identified 73 overlapping genes between ATBC exposure and bone cancer, subsequently prioritizing STAT3, EGFR, MMP9, MAPK1, and MMP2 as core targets. These findings reveal a previously underappreciated carcinogenic risk associated with ATBC and provide new mechanistic insights into how environmental toxicants may drive tumorigenesis through signal transduction, metabolic adaptation, and microenvironmental remodeling.

The centrality of STAT3 within the interaction network underscores its pivotal role in linking ATBC exposure to the pathogenesis of bone cancer. As a transcription factor that regulates apoptosis, proliferation, and immune evasion, the activation of STAT3 has been implicated in the aggressiveness of osteosarcoma and its resistance to therapy (15–19). Our molecular docking simulations revealed that ATBC interacts with the active domain of STAT3 through hydrogen bonds and hydrophobic interactions, suggesting that ATBC may enhance STAT3 activity and consequently upregulate its downstream oncogenic targets. This mechanism, previously unexplored in relation to ATBC, implies that ATBC could directly affect transcriptional regulators involved in the pathogenesis of bone cancer. In particular, this effect could be significant in cases where classical mutations are absent, highlighting the potential for environmental factors like ATBC to play a direct role in tumorigenesis. Furthermore, STAT3 is implicated not only in tumor initiation but also in metastasis, as it regulates pathways that promote cancer cell migration and immune evasion. These findings suggest that ATBC exposure may provide a significant, albeit overlooked, pathway for bone cancer development.

Similarly, EGFR and MAPK1 (ERK2), which are key regulators of the MAPK/ERK pathway, have emerged as high-priority targets. The MAPK cascade is a well-established driver of proliferation and metastasis in osteosarcoma (20–22), and our KEGG enrichment analysis confirmed that ATBC targets are significantly enriched in the MAPK signaling pathway and in pathways related to metabolic reprogramming, which are often upregulated in cancer to support rapid cell growth (23, 24). Molecular docking simulations revealed that ATBC interacts with critical residues in the EGFR kinase domain, potentially disrupting its auto-inhibitory conformation and promoting its activation. Molecular docking simulations revealed that ATBC interacts with critical residues in the EGFR kinase domain, potentially disrupting its auto-inhibitory conformation and promoting its activation. In addition, ATBC binds to MAPK1 at catalytic residues such as LYS117 and TYR30, further suggesting that ATBC may mimic endogenous ligands or block the binding of natural substrates, leading to sustained activation of the MAPK signaling axis. This finding aligns with previous studies on other plasticizers like phthalates, which have been shown to activate the EGFR/MAPK pathway in breast cancer models (25, 26). However, our study extends this mechanism to ATBC and bone cancer, proposing that ATBC may induce tumorigenic signaling through the same pathway.

The inclusion of MMP9 and MMP2 (matrix metalloproteinases) as core targets underscores the potential of ATBC to remodel the tumor microenvironment (27–29). Matrix metalloproteinases facilitate the degradation of the extracellular matrix, which enables tumor invasion and angiogenesis (29–31). Our Gene Ontology (GO) enrichment analysis indicates that the targets of ATBC are significantly associated with biological processes such as “tissue remodeling” and “cell-matrix adhesion,” both of which are directly influenced by MMPs. Matrix metalloproteinases (MMPs) play a crucial role in enabling cancer cells to invade adjacent tissues and form secondary tumors, positioning them as key mediators in the metastatic cascade. Docking simulations have demonstrated that ATBC binds to the catalytic domains of MMP2, specifically interacting with critical residues such as HIS121 and LEU138. This interaction implies that ATBC may enhance MMP2 activity or upregulate its expression through upstream signaling pathways, including STAT3. Consequently, ATBC could potentially facilitate both the degradation of the extracellular matrix (ECM) and the migration of tumor cells, which are vital processes in cancer progression. The dual function of ATBC in modulating intracellular oncogenic signaling and extracellular matrix remodeling underscores its potential contribution to the invasive and metastatic traits of bone cancer.

Notably, the KEGG pathway analysis revealed significant enrichment in hypoxia-related pathways and cancer metabolic reprogramming, both of which are hallmark features of aggressive tumors. In hypoxic microenvironments, cancer cells frequently shift their metabolic pathways toward glycolysis and other processes to sustain survival and growth (32). This metabolic reprogramming is closely associated with increased tumor aggressiveness and resistance to treatment (33–35). Previous studies have demonstrated that ATBC can induce lipid accumulation and disrupt normal metabolic pathways in liver cells (5, 36, 37). Our findings extend this concept by suggesting that ATBC may similarly assist cancer cells in adapting to metabolic stress, including hypoxic conditions. This dual impact on both genetic and metabolic axes highlights the complexity of ATBC’s carcinogenic potential (38).

This study, while providing valuable insights into the potential carcinogenic mechanisms of acetyl tributyl citrate (ATBC) through network toxicology and molecular docking, has several limitations. Initially, we employed molecular docking to predict the interaction between ATBC and the target protein. While this method offers substantial insights, the absence of molecular dynamics simulations, due to computational constraints, precludes a more nuanced understanding of their interactions, rendering it impossible to elucidate the dynamic changes in protein-ligand interactions over time. Furthermore, this study predominantly relies on molecular docking and network toxicology analysis for inference, lacking validation through *in vitro* or *in vivo* experiments. Additionally, we concentrated on protein-ligand interactions and neglected other potential mechanisms, such as non-coding RNA, RNA methylation, or post-translational modifications, which may significantly influence the action of ATBC. Moreover, our research is based on publicly available databases, which may present limitations regarding data quality and completeness. Finally, environmental factors, such as diet, air pollution, and genetic susceptibility, could impact the toxicity of ATBC, a consideration that was not addressed in this study.

Future studies should integrate molecular dynamics simulations to investigate the temporal dynamics and stability of ATBC-protein interactions, thereby enhancing our understanding of how these interactions evolve over time and under varying physiological conditions. Additionally, *in vitro* studies utilizing osteosarcoma cell lines and *in vivo* models of ATBC exposure could validate the predicted molecular interactions and their implications for cancer progression. These investigations will help bridge the gap between computational predictions and biological realities. Furthermore, a comprehensive examination of epigenetic mechanisms, including non-coding RNA, RNA methylation, and post-translational modifications, is essential to fully elucidate the complex molecular pathways through which ATBC may influence bone cancer. The integration of multi-omics data can yield a more holistic understanding of ATBC toxicity. Clinical and epidemiological studies are also warranted to evaluate the long-term effects of ATBC exposure on humans, particularly concerning the risk of bone cancer. Such research may clarify whether prolonged exposure to ATBC correlates with an increased cancer risk. Lastly, by investigating the influence of environmental factors such as diet, air pollution, and genetic predisposition on ATBC toxicity, we can achieve a more nuanced understanding of how environmental pollutants contribute to cancer risk, thereby providing critical insights into the broader implications of ATBC exposure.

## 5 Conclusion

This study elucidates the potential mechanisms of Acetyl Tributyl Citrate (ATBC) in bone cancer through an integrated approach that combines network toxicology and molecular docking. By identifying key molecular targets such as STAT3, EGFR, MAPK1, MMP9, and MMP2, the research indicates that ATBC may promote carcinogenesis by activating signaling pathways associated with proliferation, survival, and invasion. These findings suggest that exposure to ATBC could enhance the aggressiveness of bone cancer through interactions with signaling networks involved in tumor progression. Furthermore, molecular docking results confirm stable binding interactions between ATBC and these core proteins, underscoring their critical role in ATBC’s oncogenic effects. The ability of ATBC to modulate the tumor microenvironment, particularly through processes such as tissue remodeling and activation of survival pathways, offers new insights into the environmental risks linked to plasticizer exposure. Given the widespread use of ATBC and its potential carcinogenic effects, it is imperative for future research to further investigate the long-term implications of environmental pollutants, such as ATBC, on cancer development. Our findings underscore the necessity for enhanced regulatory scrutiny of environmental chemicals and their impact on public health. This study not only deepens our understanding of the molecular mechanisms of ATBC but also contributes to the broader field of environmental toxicology, highlighting the importance of examining the cumulative effects of environmental exposures on cancer etiology.

## Data Availability

The original contributions presented in the study are included in the article/supplementary material, further inquiries can be directed to the corresponding authors.

## Footnotes

^1^ https://pubchem.ncbi.nlm.nih.gov/

^2^ https://www.ebi.ac.uk/chembl/

^3^ http://swisstargetprediction.ch/

^4^ http://targetnet.scbdd.com/

^5^ https://www.uniprot.org/id-mapping

^6^ https://www.genecards.org/

^7^ https://omim.org/

^8^ https://db.idrblab.net/ttd/

^9^ https://cn.string-db.org/

^10^ https://cytoscape.org/

^11^ https://davidbioinformatics.nih.gov/

^12^ https://www.bioinformatics.com.cn/

^13^ https://www.rcsb.org/
